# Functional microRNA binding site variants

**DOI:** 10.1002/1878-0261.12421

**Published:** 2018-12-26

**Authors:** Ye Yuan, Joanne B. Weidhaas

**Affiliations:** ^1^ Department of Radiation Oncology UCLA Los Angeles CA USA

**Keywords:** biomarkers, functional variants, *KRAS‐*variant, microRNA binding sites, microRNAs, polymorphisms

## Abstract

Germline single nucleotide polymorphisms are one of the most common genetic variations. Polymorphisms that cause nonsynonymous mutations in gene coding regions are known to cause serious deleterious downstream effects. However, even polymorphisms in noncoding regions can have profound functional consequences by disrupting essential regulatory sites. Specifically, polymorphisms that alter microRNA binding sites can disrupt the regulation of hallmark biological pathways implicated in tumorigenesis and tumor progression. Many of these microRNA‐associated polymorphisms (miR‐SNPs) have recently been shown to be important biomarkers of cancer risk, prognosis, and treatment outcomes. This review will summarize the functional impact of key miR‐SNPs and define a subset of miR‐SNPs that may be clinically useful prognostic or predictive biomarkers.

**Abbreviations**
3′‐UTR: 3′‐untranslated region
5‐FU: 5‐fluorouracil
ADT: androgen deprivation therapy
BER: base excision repair
CSS: cancer‐specific survival
DSB: double‐strand break repair
ER: estrogen receptor
GWAS: genomewide association studies
HR: homologous recombination
miRNAs: microRNAs
miR‐SNP: microRNA‐associated single nucleotide polymorphism
MTX: methotrexate
NHEJ: nonhomologous end joining
SNP: single nucleotide polymorphism

## 1 Introduction

Single nucleotide polymorphisms (SNPs) are the most common source of variation within human genomes, and there are currently over 80 million mapped SNPs (1000 Genomes Project Consortium, 2015). In some cases, SNPs in coding regions of oncogenes or tumor suppressor genes can lead to gain‐of‐function or loss‐of‐function mutations resulting in malignant transformation. Although this association of functional SNPs in gene coding regions with cancer is well known, individuals who harbor these specific mutations represent an extremely small proportion of cancer patients.

Single nucleotide polymorphisms are found throughout the genome, and studies have predicted that the majority of disease‐associated SNPs reside in noncoding regions (Tak and Farnham, 2015; Yao *et al*., 2014). Genomewide association studies, or GWAS, which were the first approach to try to identify germline disease‐associated SNPs, appear to have difficulty capturing clinically relevant noncoding region SNPs, perhaps in part due to the complexity of accurate annotation (Nishizaki and Boyle, 2017), or limitations in SNP inclusion due to platform restraints. However, subsequent direct experimental testing of noncoding region SNPs has shown they can have significant functional effects on gene expression by disrupting transcriptional regulatory sites (Kasowski *et al*., 2010; Maurano *et al*., 2012) or altering the binding of other recently discovered regulatory factors such as microRNAs (miRNAs) (Saunders *et al*., 2007). miRNAs are short 18‐ to 24‐nucleotide RNA molecules that play an important role in regulating many biologic pathways including pathways involved in cancer progression (Caldas and Brenton, 2005; Calin and Croce, 2006; Ceppi and Peter, 2014; Kent and Mendell, 2006; Kong *et al*., 2012; Lujambio and Lowe, 2012). They exert their regulatory control by binding via complete or partial complementarity with sequences in the 3′ UTR of a target mRNA. This subsequently results in silencing of gene expression through either sequestration or degradation of the target mRNA. Slight changes in the miRNA binding sequence in the 3′ UTR can change miRNA and mRNA binding leading to alterations of these key regulatory interactions (Saunders *et al*., 2007) so that even single nucleotide changes introduced by germline polymorphisms within miRNA binding sites (miR‐SNPs) can have profound downstream effects. The aims of this review were to (a) define the biological effects of miR‐SNPs, (b) distinguish between prognostic and predictive miR‐SNP‐based biomarkers, and (c) provide clinically promising examples of each.

### 1.1 Alterations of cancer pathways by miR‐SNPs

Hanahan and Weinberg proposed that cancer develops and progresses through aberrations in key biological pathways and there is strong evidence that miRNAs are key players in maintaining these hallmark pathways (Hanahan and Weinberg, 2011). Aberrations in miRNA expression or alterations in their binding can lead to tumorigenesis and cancer progression via one of these canonical pathways. For instance, the miR‐15 and miR‐16‐1 family of miRNAs downregulate the expression of the anti‐apoptotic protein *BCL2* and loss of these two miRNAs leads to the development of B‐cell chronic lymphocytic leukemia (Fabbri *et al*., 2009). Several SNPs have been identified in the noncoding regions of *BCL2* including rs1564483 (G>A) a functional variant in the *BCL2* 3′ UTR that is associated with decreased risk for non‐small‐cell lung cancer (NSCLC) (Xu *et al*., 2013) while yet another variant rs2279115 (C>A) appears to increase risk of esophageal SCC (Pan *et al*., 2015).

Similarly, unfettered activation of proliferative pathways is central to malignant transformation. For example, mutated *KRAS* leads to constitutive activation of proproliferative signaling pathways downstream of *EGFR* and is implicated in a significant proportion of colorectal cancers (Normanno *et al*., 2009). However, even in *KRAS* wild‐type patients, a germline variant in the let‐7 miRNA binding site of the *KRAS* 3′ UTR (rs61764370, *KRAS*‐variant) has been shown to increase risk for certain types of cancers and to predict treatment outcomes.

Functional variants have also been uncovered in genes involved in cell cycle progression and DNA repair pathways. *XRCC1* is a DNA repair pathway gene involved in single‐strand repair that harbors a miR‐SNP. Bioinformatic screens uncovered a functional variant, rs1799782 (C>T), within the 3′ UTR of *XRCC1*, a key gene in the DNA single‐strand break repair pathway, and gene reporter assays confirmed that this variant strengthens the binding of miR‐138. This resulted in higher *XRCC1* expression in the presence of miR‐138 (Nicoloso *et al*., 2010). These results highlight the fact that nucleotide changes introduced by miR‐SNPs can also create new binding sites for miRNAs leading to previously unforeseen regulatory interactions.

Germline mutations in coding regions of the *BRCA1* gene greatly increase the risk for hereditary breast and ovarian cancers due to defective double‐stranded DNA damage repair pathways. A functional variant, rs799917 (C>T) in an intron of the *BRCA1* coding sequence, was found to be associated with increased breast cancer risk (Nicoloso *et al*., 2010). Interestingly, the authors found that rs799917 resided in a binding site for miR‐638 within the *BRCA1* coding sequence and that the minor allele (T) diminished miR‐638's ability to repress *BRCA1* gene expression. These seemingly contradictory findings highlight the sometimes heterogeneous effects of miRNAs. In fact, miRNA binding can lead to transcriptional activation in cell type‐ and cell cycle‐dependent contexts (Shobha Vasudevan *et al*., 2008; Shohba Vasudevan *et al*., 2007).

Given the involvement of miR‐SNPs in disrupting the regulation of hallmark tumorigenic pathways, it is not surprising that there is increasing research on how miR‐SNPs relate to cancer risk and prognosis. Many studies have established a link between functional miR‐SNPs and increased risk for a variety of cancer types, and this has been recently reviewed in depth (Cipollini *et al*., 2014; Moszyńska *et al*., 2017). For the purposes of this review, we will focus specifically on functional miR‐SNPs that may develop into biomarkers that can help clinicians select the optimal treatment for cancer patients. Thus, we must distinguish between biomarkers that are prognostic versus those that are predictive. Prognostic biomarkers are genetic or genomic variations that are associated with certain clinical outcomes regardless of the selected treatment regimen. Predictive biomarkers, on the other hand, can potentially identify what subset of patients may have better outcomes from one type of treatment versus another. With this distinction in mind, we will discuss some promising miR‐SNP‐based biomarkers under development.

### 1.2 miR‐SNPs as prognostic biomarkers

Much work has been done to find miR‐SNPs that are prognostic in cancer patients. In particular, we will focus on specific variants that are prognostic for outcomes after treatment with chemotherapy, radiation, or targeted agents.

Several studies have investigated the association between miR‐SNPs and survival after chemotherapy. Wynendaele and colleagues found a variant, rs4245739 (A>C), in the 3′ UTR of *MDM4* that led to the creation of a binding site for miR‐191 and resulted in transcriptional repression of *MDM4* with the AC and CC alleles. *MDM4* is an oncoprotein that represses the activity of p53, and the authors found that in patients with ovarian cancer the A‐allele *MDM4* was associated with better median overall survival versus the miR‐191‐associated C‐allele, especially for women with ER‐negative tumors. Furthermore, patients with the *MDM4* A‐allele were at increased risk for relapse following chemotherapy (Wynendaele *et al*., 2010). Another case–control study in ovarian cancer patients found 24 miR‐SNPs associated with ovarian cancer survival and 17 miR‐SNPs that were prognostic of treatment outcome. Of these, the rs1425486 (G>A) variant in the 3′ UTR of *PDGFC* was the most prognostic and disrupted a binding site for miR‐425 (Liang *et al*., 2010).

DNA repair pathways are important for cell survival in response to therapeutic doses of ionizing radiation, and DNA repair genes are often dysregulated in cancer cells. A screen of miR‐SNPs within 20 genes involved in DNA repair pathways including base excision repair (BER), nucleotide excision repair, nonhomologous end joining (NHEJ), homologous recombination (HR), and double‐strand break repair (DSB) revealed 7 miR‐SNPs in *LIG3*,* ATM*,* BRCA1*,* PARP1*,* NBS1*, and *RAD51* of which the *RAD51*‐associated variant rs7180135 (A>G) was prognostic for 5‐year cancer‐specific survival (CSS) following radiation in patients with muscle‐invasive bladder cancer (Teo *et al*., 2012). Bioinformatic analyses of the rs7180135 site revealed a potential binding site for miR‐197 that is weakened by the G‐allele.

Finally, there are also miR‐SNPs that are prognostic for treatment outcomes following targeted therapies. A case–control study of prostate cancer patients receiving androgen deprivation therapy (ADT) uncovered a germline variant signature consisting of three (rs6728684/*KIF3C*, rs3737336/*CDON*, rs1045747/*IFI30*), four (rs6728684/*KIF3C*, rs1071738/*PALLD*, rs998754/*GABRA1*, rs4351800/*SYT9*), and one (rs4351800/*SYT9*) miR‐SNPs that were significantly correlated for disease progression, prostate cancer‐specific mortality, and all‐cause mortality, respectively. Interestingly, the multivariant signatures showed significant gene‐dosage effect with worsening prognosis in patients with increasing numbers of variants (Bao *et al*., 2011).

### 1.3 miR‐SNPs as predictive biomarkers

Predictive biomarkers can be used to classify patients based on their expected response to one treatment versus another. The presence of somatic *BRAF* V600 mutations, for instance, is a clinically significant predictive biomarker for response to small‐molecule inhibitors of *BRAF* in patients with metastatic melanoma (Chapman *et al*., 2011; Hauschild *et al*., 2012). In contrast to many prognostic biomarkers, predictive biomarkers can have direct clinical utility and can be used to select between treatment regimens. However, discovery of such predictive biomarkers requires careful study design to develop and ultimately validate a potential signature. Since the functional consequences of miR‐SNPs have only recently been appreciated, there are comparatively few germline miR‐SNPs that have been demonstrated to be predictive biomarkers at this time.

A few miR‐SNPs have shown potential as predictive markers in preclinical studies. Rs34764978 is a variant in the 3′ UTR of *DHFR*, a critical gene in purine biosynthesis that is targeted by the chemotherapy agent methotrexate (MTX). This variant also appears to disrupt the binding site for miR‐24 resulting in higher expression *DHFR* in variant‐harboring cells (Mishra *et al*., 2007). The authors found that *DHFR* levels were higher in cells with the rs34764978 variant in the presence of miR‐24 and were more resistant to treatment with MTX. Additional follow‐up studies, including well‐controlled clinical studies, will be needed to determine the predictive power of the rs34764978 variant in patients who receive MTX versus those who do not. In another study, Pardini and colleagues looked at miR‐SNPs in genes of the BER pathway to determine whether any would be prognostic for colorectal patients treated with 5‐fluorouracil chemotherapy (5‐FU). They hypothesized that since BER is the predominant mechanism for repairing 5‐FU induced DNA lesions, alterations of BER pathway genes by miR‐SNPs would be important. One variant, rs2233921 (G>T), was indeed predictive for patients who were homozygous for the T‐allele and received 5‐FU showing the best survival (Pardini *et al*., 2013).

Currently, the *KRAS*‐variant is a miR‐SNP with the best clinical evidence as a predictive biomarker. Recently, the impact of the *KRAS*‐variant on treatment outcomes was analyzed in a secondary analysis of a large multi‐institutional randomized trial (Weidhaas *et al*., 2017). This trial, NRG Oncology RTOG 0522, randomized 891 patients with locally advanced oropharyngeal HNSCC between the standard of care of cisplatin‐based chemoradiation or cisplatin‐based chemoradiation with the anti‐*EGFR* monoclonal antibody, cetuximab. Of the 70 patients found to have the *KRAS*‐variant, the addition of cetuximab to cisplatin and radiation significantly increased both progression‐free survival (PFS) and overall survival (OS). Further validation of the *KRAS*‐variant's efficacy as a predictive biomarker in a dedicated clinical trial of this regimen that randomizes patients into treatment groups based on *KRAS*‐variant status is currently planned.

## 2 Discussion

miRNAs are noncoding RNAs that post‐transcriptionally regulate much of the coding genome. Disruptions in miRNA::mRNA regulatory interactions are known to lead to tumorigenesis and cancer progression. Germline variants in conserved miRNA binding sites, known as miR‐SNPs, have recently been shown to play an important role in pathogenic alterations of miRNA regulatory networks including those that modulate hallmark tumorigenic pathways.

A more complete molecular understanding of how miR‐SNPs alter miRNA regulatory networks is still needed. In particular, variations in miRNA binding sites can lead to a range of effects on miRNA::mRNA interactions, from complete disruption of binding to the creation of a new miRNA binding site. While *in silico* prediction software can be useful in screening for miR‐SNPs and effected miRNAs, gene reporter assays remain the gold standard for experimentally verifying functional variants. Interpreting the effects of miR‐SNPs on biological pathways can sometimes be complicated by the fact that miRNAs can have cell type‐dependent and cell cycle‐dependent effects on target mRNAs. Furthermore, because miR‐SNPs are germline variants present in both normal host and malignant cells it is important to consider the effects of these polymorphisms on both tumor cells and peritumoral normal cells.

While many questions still remain regarding the downstream biological effects of miR‐SNPs, there is much work being done to see whether these germline polymorphisms can be used to risk‐stratify cancer patients. Especially with improvements in the efficiency and cost of DNA sequencing technology, screening for miR‐SNPs can potentially be easily integrated into the clinical workflow with potentially far‐reaching clinical application. Many miR‐SNPs have already been shown to be useful in a variety of cancer types as prognostic biomarkers. However, there are still relatively few predictive miR‐SNP‐based biomarkers that can help clinicians and patients personalize treatment decisions. Development of such predictive miR‐SNP biomarkers will require careful patient selection and validation with clinical trials that randomize patients into treatment groups based on their biomarker status.

## Conflicts of interest and disclosure

Weidhaas is an inventor on a patent filed by Yale University regarding the *KRAS‐*variant and the founder of a company that has licensed the patent from Yale University. Yuan has no conflicts.

## Author contributions

YY and JBW wrote the manuscript.
